# Characterizing Inhibitory Control Challenges Among Autistic Adults: An Examination of Demographic and Psychiatric Moderators and Associations with Anxiety Symptomatology

**DOI:** 10.1007/s10803-025-06799-8

**Published:** 2025-05-17

**Authors:** Meghan K. O’Brien, Goldie A. McQuaid, Jessica R. McNulty, Gregory L. Wallace, Nancy Raitano Lee

**Affiliations:** 1 Department of Psychological and Brain Sciences, Drexel University, Philadelphia, PA USA; 2 Department of Psychology, George Mason University, Fairfax, VA USA; 3 Department of Speech, Language and Hearing Sciences, The George Washington University, Washington, DC USA; 4 Department of Psychology, University of Nebraska-Lincoln, Lincoln, USA

**Keywords:** Autism, Adult outcomes, Inhibitory control, Anxiety, ADHD

## Abstract

Autism spectrum disorder is associated with elevated rates of mental health difficulties and executive function challenges. Emerging evidence links executive function to mental health in autistic individuals. However, less is known about (a) everyday inhibitory control difficulties among autistic adults; (b) the influence of sex assigned at birth and co-occurring attention deficit/hyperactivity disorder (ADHD) features on inhibition challenges; and (c) relations between inhibition challenges and anxiety symptoms. Drawing upon data from 732 autistic adults aged 18–83, this online study examined self-reported inhibitory control in autistic adults and the influence of assigned sex and ADHD screening status (based on a positive screening score on a self-report tool) on the degree of inhibitory control challenges experienced. In addition, this research examined relations between inhibitory control challenges and anxiety symptoms, and the moderating role of assigned sex and ADHD screening status in this relationship. Autistic adults endorsed significantly more inhibitory control challenges relative to published norms. Participants assigned female reported more difficulties in inhibitory control relative to sex-adjusted normative expectations than participants assigned male. Participants who screened positive for ADHD reported more inhibitory control challenges than those who screened negative. Greater endorsement of inhibitory control challenges was associated with greater anxiety symptomatology; this relationship was moderated by ADHD screening status, but not by assigned sex. Inhibitory control is an area of difficulty in autistic adults and is associated with anxiety symptomatology, suggesting that inhibitory control may be a valuable intervention target to improve emotional well-being in autistic adults.

Emerging research suggests that autistic^1^ adults experience sub-optimal outcomes in the areas of employment and independent living (Billstedt et al., 2011; Roux, 2015), quality of life (Dijkhuis et al., 2017), and adaptive functioning (Nyrenius & Billstedt, 2020). While research indicates variability in outcomes among autistic adults in areas such as psychosocial and vocational functioning (Howlin & Magiati, 2017; Magiati et al., 2014; Scheeren et al., 2022), research examining the individual factors contributing to these differences in adult outcomes remains limited.

One factor that may relate to this variability is the presence of co-occurring psychiatric conditions. While estimated rates of mental health conditions in autism are variable (some reviews have estimated that rates of comorbid psychiatric disorders are around 54–57%, while others estimate as many as 86% of autistic adults have at least one mental or behavioral comorbidity; Howlin & Magiati, 2017), there is evidence that autism commonly co-occurs with several psychiatric and neurodevelopmental conditions, including anxiety, depression, and ADHD (Howlin & Magiati, 2017). Research has shown that the rates of anxiety and other mental health conditions are significantly greater in autistic adults relative to the non-autistic population (Gotham et al., 2015; Hollocks et al., 2019; Howlin & Magiati, 2017; Kilanko et al., 2022; Nimmo-Smith et al., 2020). Despite this, there is only a small body of research examining factors that predict or are related to mental health challenges among autistic adults. One factor that may be of particular relevance, and that is therefore the focus of the current study, is executive function challenges. Executive function challenges are well documented in this population, particularly in the domain of cognitive flexibility (McEvoy et al., 1993; Mihailescu et al., 2022; St John et al., 2022; Xie et al., 2020). The research investigating the executive function subdomain of inhibitory control challenges, however, has focused more on autistic children. Further, the small body of existing research on inhibitory control in autistic adults reveals disagreement in results (St John et al., 2022). The following sections summarize the literature that has investigated (a) whether autistic adults present with greater inhibitory control challenges than neurotypical peers, and (b) relationships between executive function challenges, including inhibitory control difficulties, and everyday outcomes, including mental health challenges.

## Inhibitory Control Among Autistic Adults and Neurotypical Peers

A Web of Science search using the search terms autis* OR asperger* (Title) and inhib* (Abstract) and adult* (Abstract) yielded 301 results. An additional 23 studies were identified from a recent review of executive function in autistic adults (St John et al., 2022). Of the 324 studies identified, 42 were determined to be relevant to the present research question. The 42 studies identified by this search that have examined inhibitory control among autistic adults using either performance-based measures or informant report tools are summarized in Table 1. To be included in this table, the study needed to include (a) a group of autistic adults (i.e., not a group with both children and adults), and (b) a neurotypical comparison group or normative data to serve as a source of comparison for the autistic group. Of the identified studies employing performance-based measures, 41% observed a statistically significant disadvantage in inhibitory control among autistic adults compared to neurotypical adults (e.g., Johnston et al., 2019; Mihailescu et al., 2022; Uzefovsky et al., 2016). While several studies found no significant differences between autistic groups and control groups, a few important trends emerged.

**Table 1:** Review of studies examining inhibitory control in autistic adults

Study	AUT age mean (range)	NT age mean (range)	AUT Sample M: F	NT Sample M: F	Inhibition measure		Sig. findings	Notes
	*Studies employing performance-based measures of inhibition*							
Altgassen and Koch, (2014)	25.82 (SD 7.08)	25.64 (SD 5.44)	20:2	20:2	Go/No-Go Task		AUT ~ NT	Asperger’s or ASD w/o ID; no history of other psychiatric conditions
Altgassen et al., (2012)	21.8 (SD 6.68)	21.8 (SD 6.06)	20:5	19:6	Go/No-Go Task		AUT ~ NT	Asperger’s or ASD w/o ID; no history of other psychiatric conditions
Ambery et al. (2006)	37.6 (19–67)	33.5 (21–58)	22:5	16:4	Stroop Test		AUT ~ NT*	ASD w/o ID, ADHD, or other psychiatric condition
Amestoy et al. (2021)	29 (SD 6.9)	29.3 (SD 6.7)	25:10	21:8	Anti-Saccade Task		AUT ~ NT	ASD w/o ID or other psychiatric condition
Baez et al. (2012)	35.46 (SD 11.86)	35.7 (SD 11.52)	11:4	11:4	Hayling Test		AUT ~ NT*	Asperger’s; IQ above 94; no history of other psychiatric condition
Bogte et al. (2008)	28 (20–39)	28 (19–39)	21:2	14:18	Sternberg RT paradigm		AUT ~ NT	ASD w/o ID only
Brady et al. (2017)	18.86 (16.3–21.5)	18.9 (16-21.5)	26:8	26:8	DKEFS Color-Word Interference Test		AUT > NT	ASD w/o ID only
Carmo and Filipe (2022)	26.43 (19–43)	27.61 (21–43)	13:1	17:1	Hayling Test		AUT ~ NT	ASD w/o ID; participants had > 9 years of formal education
Coutelle et al. (2020)	30.07 (SD 9.06)	31.05 (SD 10.36)	26:16	26:16	Hayling Test		AUT > NT	ASD w/o ID or with clinical diagnosis of MDD
Dehnavi and Khan (2024)	31.66 (25–47)	33.03 (25–55)	40:10	42:14	Stroop Test		AUT ~ NT*	
Fossum et al. (2021)	22.2 (17–27)	20.9 (17–27)	21:5	26:14	DKEFS Color-Word Interference Test		AUT > NT	ASD w/o ID only
Fried et al. (2016)	27.5 (18–40)	27.5 (21–36)	20:6	40:12	DKEFS Color-Word Interference Test		AUT > NT	ASD w/o ID only
Hill and Bird (2006)	31.09 (16–61)	33.45 (18–64)	16:6	14:8	Hayling Test, Stroop Test		AUT ~ NT*	Asperger’s only
Ishii-Takahashi et al. (2014)	30.8 (SD 7.2)	28.8 (SD 5.5)	8:13	13:8	Stop-Signal Task		AUT ~ NT*	ASD w/o ID, ADHD, or other psychiatric condition
Johnston et al. (2011)	27.8 (SD 8.7)	28.7 (SD 11.1)	19:5	10:4	Stroop Test, Hayling Test		AUT ~ NT*	ASD w/o ADHD
Johnston et al. (2019)	33.5 (SD 10.86)	30.84 (SD 7.3)	88:22	26:5	Hayling Test		AUT > NT	ASD w/o ID or other neurodevelopmental disorder including ADHD
Kana et al. (2007)	26.8 (SD 7.7)	22.5 (SD 3.2)	11:1	11:1	Letter-based inhibition task		AUT ~ NT	ASD w/o ID only
Lai et al. (2012)	27.55 (18–49)	28.15 (18–49)	32:32	32:32	Go/No-Go Task		AUT > NT	
Landsiedel and Williams (2020)	34.84 (SD 11.42)	38.24 (SD 13.19)	21:4	19:4	Stroop Test		AUT ~ NT*	
Langen et al. (2012)	25.57 (19–39)	28.45 (19–44)	21:0	21:0	Go/No-Go Task		AUT > NT	ASD w/o ID or other psychiatric condition
Lopez et al. (2005)	29.1 (19–42)	29.4 (18–45)	14:3	11:6	DKEFS California Stroop Test		AUT ~ NT*	
Luna et al. (2007)	17 (8–33)	17 (8–33)	58:3	58:3	Anti-Saccade Task		AUT > NT	ASD w/o ID only; these results represent only the adults aged 18–33
Mihailescu et al. (2022)	25.03 (16–50)	25.72 (16–56)	25:7	21:11	CANTAB multi-tasking test		AUT > NT	ASD w/o ID only
Morais et al. (2024)	22.9 (SD 4.9)	25.8 (SD 6.8)	15:0	15:0	Stroop Test		AUT > NT	
Norris and Maras (2022)	33.82 (18–58)	35.1 (18–59)	15:11:2 (gender fluid/no preference)	8:21	DKEFS Color Stroop		AUT ~ NT	
Padmanabhan et al. (2015)	24.9 (19–33)	23.4 (18–31)	8:0	12:2	Anti-Saccade Task		AUT > NT	Also found unique patterns of brain activity
Patrick et al. (2020)	19.88 (16–26)	19.79 (16–26)	40:8	39:9	Stroop Test		AUT > NT	ASD w/o history of developmental disability, ID, or psychosis (ADHD and mood disorders not excluded)
Prat et al. (2016)	25.3 (18–35)	25.6 (19–44)	10:6	11:6	Go/No-Go Task		AUT ~ NT*	
Raymaekers et al. (2004)	28.4 (SD 8.4)	28.8 (SD 8.9)	15:2	15:2	Go/No-Go Task		AUT ~ NT*	“High-functioning” autism only; task measured in context of increased arousal via fast presentation rate of stimuli
Schmitz et al. (2006)	38 (SD 9)	39 (SD 6)	10:0	12:0	Go/No/Go Task Stroop Test		AUT ~ NT	ASD w/o history of medical, psychiatric, biochemical, hematologic, or chromosomal disorders or abnormalities
Shirama et al. (2016)	26.3 (SD 1.9)	27.7 (SD 2.1)	13:3	13:3	Anti-Saccade Task		AUT ~ NT*	
Spengler et al. (2010)	35.6 (SD 12.4)	33 (SD 10.7)	12:6	12:6	Imitation/Inhibition Task		AUT > NT	
Torenvliet et al. (2023)	47.3 (20–79)	45.8 (20–77)	74:31	82:57	Go/No-Go Task		AUT ~ NT*	ASD w/o ID or history of psychosis
Towgood et al. (2009)	31.76 (19–47)	30.64 (20–43)	17:4	18:4	Hayling Test		AUT > NT	PIQ & VIQ both > 85
Uzefovsky et al. (2016)	37.15 (18–69)	38.76 (18–79)	101:100	126:114	Go/No-Go Task		AUT > NT	ASD w/o ID only
Velasquez et al. (2017)	25.84 (18–34)	29.03 (20–46)	13:6	16:6	Go/No-Go Task		AUT ~ NT*	ASD w/o ID only
Wilson et al. (2014)	26 (18–43)	28 (18–43)	89:0	89:0	Go/No-Go Task		AUT > NT	ASD w/o other psychiatric condition
Yon-Hernández et al. (2023)	29.38 (16–54)	38.48 (18–63)	Not reported	Not reported	Anti-Saccade Task Stop-Signal Task Stroop Test		AUT ~ NT*	ASD w/o ID only
Yuk et al. (2020)	26.94 (SD 6.08)	27.29 (SD 5.94)	27:13	27:12	Go/No-Go Task		AUT ~ NT	ASD w/o ID only; also found unique patterns of brain connectivity
	*Studies employing informant report measures of inhibition*							
Davids et al. (2016)	58.6 (50–84)	59.4 (50–79)	30:6	30:6	BRIEF-A Self		AUT ~ NT*	All diagnosed > 30 years old
Dijkhuis et al. (2017)	21.9 (18–38)	22.7 (18–28)	67:8	23:5	BRIEF-A Self		AUT ~ NT*	ASD w/o ID only
Landsiedel and Williams (2020)	34.84 (SD 11.42)	38.24 (SD 13.19)	21:4	19:4	BRIEF-A Self		AUT > NT	
Mihailescu et al. (2022)	25.03 (16–50)	25.72 (16–56)	25:7	21:11	BDEFS Self		AUT > NT	ASD w/o ID only
Wallace et al. (2016)	21.55 (18–40)	No control group– used norms	31:4	N/A	BRIEF-A Informant		AUT > NT	ASD w/o ID only
Yuk et al. (2020)	26.94 (SD 6.08)	27.29 (SD 5.94)	27:13	27:12	BRIEF-A Self BRIEF-A Informant		AUT ~ NT*	ASD w/o ID only

AUT > NT indicates greater impairment in inhibitory control in autistic group

*Studies that described a non-significant trend toward greater impairment in autistic group

Abbreviations: AUT = Autistic; BRIEF-A = Behavior Rating Inventory of Executive Function-Adult Version; DKEFS = Delis–Kaplan Executive Function System; ID = Intellectual Disability; NT = neurotypical; PIQ = Performance IQ; VIQ = Verbal IQ

First, several studies excluded participants with ADHD (e.g., Ambery et al., 2006; Johnston et al., 2019; and possibly others, including Langen et al., 2012 and Wilson et al., 2014, who excluded psychiatric conditions but did not specify whether ADHD was part of this exclusion), while others included a separate ADHD comparison group, excluding participants with co-occurring autism and ADHD (Ishii-Takahashi et al., 2014; Johnston et al., 2011). Only six of the identified performance-based studies reported that any participants had co-occurring autism and ADHD (Amestoy et al., 2021; Fried et al., 2016; Fossum et al., 2021; Luna et al., 2007; Patrick et al., 2020; Torenvliet et al., 2023), and only two of these investigated the influence of ADHD symptoms or a co-occurring diagnosis within the autistic group. One study found that increased ADHD symptomatology was correlated with poorer inhibitory control (Amestoy et al., 2021), while the other did not find any differences in inhibitory control based on ADHD symptoms (Torenvliet et al., 2023). Among studies with a separate ADHD comparison group, two found that participants in the ADHD group had worse inhibitory control than participants in the autistic group (Fried et al., 2016; Johnston et al., 2011), one showed differences in prefrontal brain activation associated with inhibitory control between autistic adults and adults with ADHD (Ishii-Takahashi et al., 2014), and one found no differences in inhibition between the autistic and ADHD groups (Fossum et al., 2021). The limited availability of research investigating the role of ADHD symptoms in autistic adults further motivated this study’s investigation of ADHD as a moderator of inhibitory control in autism.

The second trend that was observed was that several of the studies with null findings had small sample sizes, which limited their power to detect small but meaningful differences in performance. Indeed, despite the failure to detect statistically significant differences, the means for many of these studies suggested that autistic participants tended to perform less well on inhibitory control tasks than neurotypical participants (e.g., Ambery et al., 2006; Baez et al., 2012; Ishii-Takahashi et al., 2014; Johnston et al., 2011; Lopez et al., 2005; Velasquez et al., 2017). To better understand the findings in the literature despite the mixed results regarding statistical significance, Hedge’s *g* effect sizes were calculated, which are presented in Table 2. Studies that measured timed accuracy within the context of inhibitory control tasks showed the largest effect sizes, with an average of *g* = -0.48, indicating that the autistic groups had moderately lower timed accuracy than comparison groups. Tests that measured speed during inhibitory control tasks showed that autistic groups were slower (average *g * = -0.34). Studies that included inhibitory control accuracy as the outcome of interest showed that the autistic groups generally displayed lower accuracy, but with small effect sizes (average *g* = -0.28). Several studies measured “sensitivity” as a proxy for accuracy, which is a measure derived from signal detection theory that takes into account correct responses, or “hits,” and commission errors, or “false alarms.” The average effect size for measures of sensitivity was moderate, with *g* = -0.44. Measures of inhibitory control errors also showed that autistic groups made more errors than comparison groups with small effect sizes (average *g* = -0.21).

**Table 2:** Effect sizes in studies examining inhibitory control in autistic adults

Study	Inhibition measure of interest	Outcome (category)	Effect size of results (Hedges’ g)	Description of outcome (for AUT group)
Altgassen and Koch (2014)	**Go/No-Go** (inhibition condition), proportion of correct responses	Accuracy	0	Equivalent accuracy
Altgassen et al. (2012)	**Go/No-Go**, T-Score	Timed accuracy	-0.15	Weaker performance
Ambery et al. (2006)	**Stroop interference**, # of colors named in 120 s	Timed accuracy	-0.37	Weaker performance
Amestoy et al. (2021)	**Antisaccade task**, latency time in ms	Time	0.16	Faster
Baez et al. (2012)	**Hayling**, reaction time	Time	-0.52	Slower
Bogte et al. (2008)	**Sternberg visual memory search task** (response bias condition), errors of commission	Errors	-0.19*	More errors
Brady et al. (2017)	**DKEFS Color Word Interference** (inhibition condition), scaled score	Time	-0.69	Slower
Carmo and Filipe (2022)	**Hayling** (inhibition condition), accuracy	Accuracy	0.39	More accurate
Coutelle et al. (2020)	**Hayling**, inhibition score	Timed accuracy	-0.55	Weaker performance
Davids et al. (2016)	**BRIEF-A Self** (Inhibit subscale), T-Score	Endorsed challenges	-0.38*	More challenges
Dehnavi and Khan (2024)	**Stroop** (incongruent items), fixation duration	Fixation duration	-1.26	Longer fixation time
Dijkhuis et al. (2017)	**BRIEF-A Self** (Inhibit subscale), raw score	Endorsed challenges	-0.31*	More challenges
Fossum et al. (2021)	**DKEFS Color Word Interference** (inhibition condition), completion time in seconds	Time	-1.24	Slower
Fried et al. (2016)	**DKEFS Color Word Interference** (inhibition condition), scaled score	Time	-1.33	Slower
Hill and Bird (2006)	**Hayling** (inhibition condition), response time	Time	-0.66	Slower
	**Hayling** (inhibition condition), errors	Errors	-0.43	More errors
	**Stroop** (inhibition condition), # of colors named in 120s	Timed accuracy	-0.36	Weaker performance
Ishii-Takahashi et al. (2014)	**Stop-Signal Test** (stop trials), % correct	Accuracy	-0.51	Weaker performance
	**Stop-Signal Test** (all trials), % correct	Accuracy	-0.39	Lower accuracy
Johnston et al. (2011)	**Hayling** (inhibition condition), total errors scaled score	Errors	0.53	Fewer errors
	**Hayling** (inhibition condition), inhibition time in seconds	Time	-0.92	Slower
	**Color-word task**, standard score - # of correct colors named in 120 s minus errors	Timed accuracy	-0.50	Weaker performance
	**Color-word task**, total words	Timed accuracy	-0.51	Weaker performance
Lai et al. (2012)	**Go/No-Go** criterion – *female sample only*	Response strategy	0.31	More conservative strategy
	**Go/No-Go** sensitivity – *female sample only*	Sensitivity	-0.58	Less sensitive
	**Go/No-Go** criterion – *male sample only*	Response strategy	0.09	More conservative strategy
	**Go/No-Go** sensitivity – *male sample only*	Sensitivity	-0.92	Less sensitive
Landsiedel and Williams (2020)	**Stroop**, reaction time difference between congruent and incongruent trials	Time	-0.19	Slower
	**BRIEF-A Self** (Inhibit subscale), T-Score	Endorsed challenges	-1.02	More challenges
Lopez et al. (2005)	**California Stroop** (interference condition), # of errors	Errors	-0.04	More errors
	**California Stroop** (interference condition), time in seconds	Time	-0.52	Slower
Luna et al. (2007)	**Antisaccade task**, proportion of response inhibition errors	Errors	-0.50*	More errors
Morais et al. (2024)	**Stroop**, # of correct responses in 45s	Timed accuracy	-0.68	Weaker performance
Norris and Maras (2022)	**DKEFS Color Stroop**, normed contrast score of inhibition phase minus color naming phase	Time	-0.14	Slower
Padmanabhan et al. (2015)	**Antisaccade task**, % correct	Accuracy	-1.12*	Lower accuracy
Prat et al. (2016)	**Go/No-Go** (inhibition condition, faces as stimuli), accuracy	Accuracy	-0.13	Lower accuracy
	**Go/No-Go** (inhibition condition, letters as stimuli), accuracy	Accuracy	-0.20	Lower accuracy
	**Go/No-Go** (inhibition condition, faces as stimuli), sensitivity	Sensitivity	-0.40	Less sensitive
	**Go/No-Go** (inhibition condition, letters as stimuli), sensitivity	Sensitivity	-0.36	Less sensitive
	**Go/No-Go** (inhibition condition, faces as stimuli), response time in ms	Time	0.16	Faster
	**Go/No-Go** (inhibition condition, letters as stimuli), response time in ms	Time	0.46	Faster
Schmitz et al. (2006)	**Go/No-Go**, mean # of errors	Errors	0.04	Fewer errors
	**Stroop**, mean # of errors	Errors	-0.04	More errors
Shirama et al. (2016)	**Antisaccade task**, % errors	Errors	-0.23*	More errors
	**Antisaccade task**, latency in ms	Time	0.07*	Faster
Spengler et al. (2010)	**Imitation/Inhibition task**, reaction time	Time	-0.50*	Slower
	**Imitation/Inhibition task**, errors	Errors	-0.66*	More errors
Torenvliet et al. (2023)	**Go/No-Go**, mean RT in ms	Time	-0.18	Slower
	**Go/No-Go**, % commission errors	Errors	-0.11	More errors
Towgood et al. (2009)	**Hayling**, time	Time	-0.65	Slower
Velasquez et al. (2017)	**Go/No-Go** (inhibition condition, emotional faces as stimuli), % correct	Accuracy	-0.33	Lower accuracy
	**Go/No-Go** (inhibition condition, letters as stimuli), % correct	Accuracy	-0.22	Lower accuracy
	**Go/No-Go** (inhibition condition, emotional faces as stimuli), reaction time in ms	Time	0.28	Faster
	**Go/No-Go** (inhibition condition, letters as stimuli), reaction time in ms	Time	0.30	Faster
Wilson et al. (2014)	**Go/No-Go**, % commission errors	Errors	-0.71	More errors
Yon-Hernández et al. (2023)	**Antisaccade**,** Stop-Signal**,** Stroop**, combined inhibition domain score based on total accuracy and mean RT	Timed accuracy	-0.69	Weaker performance
Yuk et al. (2020)	**BRIEF-A Self** (Inhibit subscale), T-Score	Endorsed challenges	-0.50	More challenges
	**BRIEF-A Informant** (Inhibit subscale), T-Score	Endorsed challenges	-0.61	More challenges
	**Go/No-Go**, sensitivity	Sensitivity	0.08	More sensitive

*Indicates that effect size was estimated based on graph of results

Abbreviations: AUT = Autistic; BRIEF-A = Behavior Rating Inventory of Executive Function-Adult Version; DKEFS = Delis–Kaplan Executive Function System

Only six studies have examined inhibitory control in autistic adults through self- or informant-reported measures. In all these studies (see Table 1), the autistic group reported more challenges in inhibitory control with moderate effect sizes (average *g* = -0.56; See Table 2), though only three studies found these differences to be statistically significant (Landsiedel & Williams, 2020; Mihailescu et al., 2022; Wallace et al., 2016). The 2016 study by Wallace and colleagues was the only one to consider ADHD symptoms, though this study investigated ADHD symptoms as a moderator of the relationship between executive function challenges and adaptive functioning and comorbid depression and anxiety symptomatology, and did not consider differences in inhibition alone as a function of ADHD symptoms. There is evidence that performance-based and self-report measures assess different aspects of executive function, and that self-report may be a more sensitive measurement of everyday difficulties that would not be detected by laboratory-based measures (Dube et al., 2022; Snyder et al., 2021). As there have only been a handful of studies examining inhibitory control in autistic adults using self-report measures, the current study employed this approach in order to augment the field’s limited understanding of inhibitory control experiences in real-world settings among autistic adults.

A final observation about the studies presented in Table 1 is the preponderance of male participants (given differences in autism rates as a function of sex assigned at birth; Maenner et al., 2023). Because of known sex differences in inhibitory control in non-autistic samples (Sjoberg & Cole, 2018), inhibitory control challenges may be moderated by sex assigned at birth. However, this has not been closely investigated in studies of autistic adults. Only four of the identified studies reported on inhibitory control differences between autistic males and females (notably, only one of the studies reviewed here specified whether this construct was defined as gender identity or sex assigned at birth; Norris & Maras, 2022). Three of these found no differences between males and females (Ishii-Takahashi et al., 2014; Lai et al., 2012; Torenvliet et al., 2023), while one study found that autistic women had higher false alarm rates on a test of inhibitory control than autistic men (Uzefovsky et al., 2016). Thus, the current study also aims to explore differences in inhibitory control challenges based on sex assigned at birth.

## Relationship Between Executive Function and Mental Health Outcomes in Autism

A few studies have examined associations between executive function and mental health among autistic adults. This research has reported associations between executive function challenges and depression (Conner et al., 2023; Wallace et al., 2016), quality of life (Dijkhuis et al., 2017), adaptive functioning (Nyrenius & Billstedt, 2020), and anxiety (Wallace et al., 2016; Zimmerman et al., 2017). To identify research that specifically examined associations between inhibitory control and mental health outcomes in autistic adults, a Web of Science search was conducted with the search terms autis* or asperger* (Title) and adult* (Abstract) and mental health or anxi* or depress* or adaptive or quality of life (Abstract) and executive function* or inhib* (Abstract). This search yielded 109 results, four of which were determined to be relevant to the present research question. This research is summarized in Table 3. While only one of these studies (Nyrenius & Billstedt, 2020) found that the subdomain of inhibitory control significantly predicted outcomes, these studies consistently demonstrated associations between mental health constructs and overall executive function, as well as with the domains of flexibility, shifting, self-monitoring, emotion regulation, and metacognition (Charlton et al., 2023; Dijkhuis et al., 2017; Nyrenius & Billstedt, 2020; Wallace et al., 2016; Zimmerman et al., 2017).

**Table 3:** Review of studies examining the relationship between Inhibition and mental health outcomes in autistic adults

Study	Age mean (range)	Sample M: F	Inhibition measure	Outcome measure(s) of interest	Sig. associations with inhibition
Charlton et al. (2023)	52.05 (40–83)	163:224	BDEFS Self-Report	Depression	None found
Dijkhuis et al. (2017)	22.12 (18–28)	67:8	BRIEF-A Self-Report	Quality of life	None found*
Nyrenius and Billstedt (2020)	21.6 (18–30)	21:9	DKEFS Color-Word Interference Test	Adaptive functioning	(+)
Zimmerman et al. (2017)	34.02 (18–66)	27:15	Hayling test	Anxiety, depression, stress	None found*

(+) Indicates that greater impairment in inhibition is associated with greater challenges in mental health outcomes

*Studies that described a non-significant trend toward a (+) relationship

Abbreviations: BRIEF-A = Behavior Rating Inventory of Executive Function-Adult Version; DKEFS = Delis–Kaplan Executive Function System

To date, only one study has examined the association between the current study’s variables of interest (inhibitory control and anxiety; Zimmerman et al., 2017). While this study did not find a significant relationship between inhibition challenges and anxiety, a small, non-significant trend was found such that lower scores on the inhibitory control measure (reflecting slower response latency and greater errors) were associated with greater endorsement of anxiety symptoms. Notably, none of the studies that utilized self- or informant-report measures of executive functions among autistic adults examined relations between everyday inhibitory control challenges and anxiety symptomatology. Thus, the goal of this study is to expand our current understanding of inhibitory control in autistic adults and explore its association with mental health outcomes through the examination of self-reported challenges in inhibitory control and generalized anxiety disorder symptoms.

In particular, the current study has four aims: (1) characterize self-reported inhibitory control challenges in a large sample of autistic adults relative to published norms; (2) explore whether difficulties with inhibitory control in this sample vary as a function of sex assigned at birth or ADHD screening status; (3) evaluate whether there is a significant relationship between inhibitory control challenges and anxiety symptomatology; and (4) assess whether sex assigned at birth or ADHD screening status are moderators of any relationship between inhibitory control and anxiety.

Given the existing literature that suggests challenges in multiple domains of executive function in autistic individuals throughout both childhood and adulthood (Xie et al., 2020), it was hypothesized that autistic adults would report significant challenges in inhibitory control. It was also hypothesized that assigned males would demonstrate greater inhibitory control challenges than assigned females. This hypothesis was based on the observed sex differences in inhibitory control in neurotypical adults (Sjoberg & Cole, 2018). Additionally, due to the characteristic challenges with inhibitory control that are reported in ADHD (Chamorro et al., 2022; Willcutt et al., 2005), it was hypothesized that autistic adults who screened positive for ADHD would endorse more inhibitory control challenges than those who screened negative.

The existing literature also suggests that executive dysfunction is associated with challenges in mental health; for this reason, it was hypothesized that greater endorsement of inhibitory control challenges would be associated with greater self-reported anxiety symptoms. As above, it was hypothesized that the relationship between inhibitory control and anxiety would be moderated by both sex assigned at birth and ADHD screening status, as these factors are known to be related to both inhibitory control (Chamorro et al., 2022; Sjoberg & Cole, 2018; Willcutt et al., 2005) and anxiety symptoms (Bangasser & Cuarenta, 2021; Kessler et al., 2006; Van Ameringen et al., 2011).

## Method

This research drew upon data obtained via a larger study of adult outcomes in autism collected between December 2019 and mid-January 2020. Prior to completing study procedures, informed consent was obtained from all participants, consistent with guidelines in the Declaration of Helsinki. Participants completed study procedures online and were compensated for their time with a $25 gift card.

### Participants

A total of 732 autistic adults (59.6% assigned female sex at birth) between 18 and 83 years of age were included in the current investigation (see Table 4 for details). They were recruited via the Simons Powering Autism Research (SPARK; Spark Consortium, 2018) Research Match service (Project No. RM0045Wallace; see Yerys et al., 2022 for additional information) which uses a multimodal approach to participant recruitment, with centralized recruitment via an online platform as well as recruitment via SPARK “clinical sites”. For more information, see Daniels et al. (2023). All study procedures were approved by the George Washington University IRB. Participants independently elected to participate in the study and provided informed consent. These participants were a part of SPARK’s “independent” adult cohort, in which all participants are at least 18 years of age, do not have a court-appointed legal guardian, and are able to consent to research participation independently. No participants self-reported a diagnosis of intellectual disability in the study’s medical history form. To be included in the larger study, participants were required to report an autism spectrum diagnosis made by a licensed professional. While the study did not confirm diagnostic status, SPARK’s partnership with autism clinical sites increases the likelihood that participants have a professionally assigned autism diagnosis. Additionally, one previous study, using information from participants’ electronic medical records, confirmed that 98.8% of a sample of SPARK participants had a documented autism spectrum diagnosis (Fombonne et al., 2022).

**Table 4:** Current study: participant characteristics

	*N* = 732
**Age, years**	
Mean (SD) Median (Range)	39.76 (13.56) 38.21 (18.17–83.33)
**Birth-sex**, ***n*(%)**	
Female Male	436 (59.56%) 296 (40.44%)
**Ethno-racial identity**	
*Race*,* n (%)*	
African American or Black Asian More than one race Native American/Native Alaskan Other White Not reported	17 (2.32%) 11 (1.50%) 73 (9.97%) 8 (1.09%) 14 (1.91%) 607 (82.92%) 2 (0.27%)
*Ethnicity*,* n (%)*	
Hispanic/Latinx Not Hispanic/Latinx Unknown Not reported	61 (8.33%) 657 (89.75%) 10 (1.37%) 4 (0.55%)
**Autism Quotient**	
Above cut-off score, *n (%)*	693 (94.7%)
**BDEFS self-restraint**	
Mean (SD) Median (Range)	39.92 (11.39) 38 (19–76)
**GAD-7**	
Mean (SD) Median (Range)	10.01 (6.31) 9 (0–21)
*GAD-7 Severity level*,* n (%)*	
None (0) Minimal (1–4) Mild (5–9) Moderate (10–14) Severe (11–21)	43 (5.87) 128 (17.49) 204 (27.87) 147 (20.08) 210 (28.69)
**ASRS**	
Screen Positive, *n (%)* Screen Negative, *n (%)*	350 (48.14%) 377 (51.86%)

Abbreviations: ASRS = Adult ADHD Self-Report Scale; BDEFS= Barkley Deficits in Executive Functioning Scale; GAD-7 = Generalized Anxiety Disorder 7-item scale

As one aim of the current research is to examine whether inhibitory control difficulties among autistic adults are elevated relative to published norms from the general population, participants with acquired neurological conditions that are likely to impact inhibitory control were excluded. Accordingly, in addition to meeting broader study inclusion criteria, participants were required to have complete data on primary variables of interest and were excluded if they reported a history of traumatic brain injury (*N* = 27), stroke (*N* = 17), Parkinson’s Disease (*N* = 2), or dementia (*N* = 3).

### Measures

The Barkley Deficits in Executive Functioning Scale (BDEFS; Barkley, 2011) Self-Restraint subscale was used to quantify participants’ inhibitory control challenges. The 19-item subscale assesses the ability to manage impulses and inhibit automatic responses. The BDEFS has demonstrated high internal consistency in the general population (Cronbach’s alpha = 0.91; Kamradt et al., 2021). The total score from the Self-Restraint scale was used for this analysis; higher scores on this measure indicate greater challenges. To compare participants’ self-reported inhibitory control challenges to normative expectations, the total score from the Self-Restraint scale was transformed into a z-score based on the published norms for this measure, which are standardized by both age and sex assigned at birth.

The Generalized Anxiety Disorder 7-item scale (GAD-7; Spitzer et al., 2006) is a self-report screening tool for identifying generalized anxiety symptoms. This scale has demonstrated high internal consistency in the general population (Cronbach’s alpha = 0.92; Spitzer et al., 2006) and among autistic adults (Cronbach’s alpha = 0.92; McQuaid et al., 2023). The item total sum was used in analyses, with greater scores indicating greater endorsement of anxiety symptoms. While participants in this study did self-report previously diagnosed psychiatric conditions, including anxiety disorders, this measure was used in analyses in order to gain more nuanced insight into self-reported anxiety symptoms and to be able to detect relationships in regression analyses by using a continuous measure of anxiety symptoms.

The Adult ADHD Self-Report Scale (ASRS; Kessler et al., 2005) was used in this study as a screener for suspected ADHD in participants. Items on this 18-item measure probe ADHD symptoms that are most representative of ADHD in adulthood. This measure has demonstrated high internal consistency (Cronbach’s alpha = 0.88; Adler et al., 2006) and high concordance with blinded clinical diagnoses of ADHD in adults (Ustun et al., 2017). The first six questions in this measure constitute an established screening tool (Kessler et al., 2005) and are considered the most predictive of symptoms consistent with ADHD (Kessler et al., 2007). These screening items were therefore used in this analysis, with participants’ scores on these items translated into a binary variable indicating a positive or negative screening. This measure was chosen to identify participants with high levels of ADHD symptomatology over participants’ self-reported ADHD diagnoses because of the well-documented disparities in ADHD diagnoses for autistic adults (Eberhard et al., 2022; Magnin & Maurs, 2017; Martin et al., 2024). Using a screener to identify participants who display clinically significant ADHD symptoms allows for the inclusion of participants who were not able to access a diagnosis. Because the focus of this investigation was on inhibitory control difficulties and we were interested in whether ADHD screening status is associated with heightened inhibitory control difficulties relative to ASD alone, item content overlap between the ASRS and Barkley scales was assessed. The purpose of this analysis was to exclude the possibility that any association found between inhibitory control and ADHD screening status was driven by the measures having overlapping item content. No concerns about item overlap between the six ASRS screening questions used in the current study and the BDEFS Self-Restraint subscale were identified.

### Statistical Analysis

Prior to conducting analyses to test study hypotheses, the data were evaluated for assumptions of parametric tests; these assumptions were satisfied. Additionally, the need to include covariates in analyses was considered. With respect to covariates, there is some evidence to suggest that there may be a significant effect of age on inhibitory control abilities in autistic individuals (such that autistic adolescents and adults do not experience the same age-related improvements as their neurotypical peers in inhibition; Padmanabhan et al., 2015; Schmitt et al., 2018); thus, age was included as a covariate to account for the large age range of the sample in primary study analyses.

Aim 1, characterizing inhibitory control challenges in the sample, was investigated using a one-sample t-test. Each participant’s total Self-Restraint z-score was compared to the population z-score mean of 0. Aim 2, exploring differences in inhibitory control challenges by sex assigned at birth and ADHD screening status, was investigated using two independent samples t-tests. The first test compared groups on the basis of sex assigned at birth (male or female), and the second compared groups on the basis of ADHD screening status (screen positive or screen negative). Both tests were computed first using the BDEFS Self-Restraint raw scores, to measure absolute differences between groups, then again using the age- and sex-normed z-scores, to compare whether the groups differed in their self-rated challenges relative to the normative sample.

Aim 3, investigating associations between inhibitory control challenges and anxiety symptoms, was investigated using a hierarchical linear regression, with age entered in the first step as a covariate, the Self-Restraint raw score entered in the second step as the independent variable of interest, and anxiety symptoms as the dependent variable. The moderation analysis, for aim 4, was also conducted as a hierarchical linear regression, with age entered in the first step as a covariate, the Self-Restraint raw score entered in the second step, assigned sex entered in the third step, and the interaction between assigned sex and Self-Restraint entered in the fourth step. Similarly, to assess the moderating role of ADHD screening status, a hierarchical linear regression was conducted, with age entered in the first step, Self-Restraint raw score entered second, the ADHD screening status variable entered third, and finally the interaction variable.

## Results

Prior to running the study’s primary analyses, correlations among the study’s primary variables of interest were investigated and are summarized in Table 5. For aim 1, characterizing self-reported inhibitory control challenges in this sample of autistic adults, z-scores significantly diverged from normative expectations, with a group mean z-score of 1.29, which was shown in a one-sample t-test to be significantly different than 0, *t*(731) = 26.68, *p* <.001. The effect size, as measured by Cohen’s *d*, was *d* = 0.99, indicating a large effect.

**Table 5:** Correlation matrix of primary study variables

Variable	*N*	M	SD	GAD-7 total	Age	Self-restraint total	Sex assigned at birth
GAD-7 total	727	10.05	6.30	--	--	--	--
Age	727	39.76	13.51	− 0.09**	--	--	--
Self-restraint total	727	39.94	11.42	0.46***	− 0.05	--	--
Sex assigned at birth (1 = female)	727	--	--	0.15***	− 0.05	− 0.02	--
Suspected ADHD status (1 = positive)	727	--	--	0.32***	− 0.01	0.40***	0.07*

*p < .05. **p < .01. ***p < .001

Abbreviations: GAD-7 = Generalized Anxiety Disorder 7-item scale

To evaluate the influence of sex assigned at birth for aim 2, we conducted an independent samples t-test. Raw Self-Restraint scores did not differ between participants assigned male at birth (*M* = 40.18, *SD* = 12.11) and participants assigned female at birth (*M* = 39.75, *SD* = 10.88), *t*(587.30) = 0.49, *p* =.63. However, an independent samples t-test using the age- and sex-normed z-scores showed a significant difference, *t*(730) = -2.56, *p* =.01, such that autistic adults assigned female (*M* = 1.39, *SD* = 1.31) reported significantly more challenges relative to the published norms adjusted for sex than autistic adults assigned male (*M* = 1.14, *SD* = 1.28), with a small effect size (Cohen’s *d* = 0.19).

Aim 2 additionally sought to evaluate the influence of ADHD screening status on inhibitory control challenges. Participants who screened positive on the ADHD rating scale endorsed significantly more inhibitory control challenges (*M* = 44.63, *SD* = 11.33) than participants who screened negative (*M* = 35.59, *SD* = 9.66) when comparing raw scores, *t*(687.92) = -11.53, *p* <.001, with a large effect size of *d* = 0.86. This difference remained significant when analyzed using the age- and sex-normed z-scores, such that participants screening positive for ADHD (*M* = 1.83, *SD* = 1.30) reported significantly more challenges than participants screening negative for ADHD (*M* = 0.79, *SD* = 1.11), *t*(689.79) = -11.66, *p* <.001. This effect size was also large, *d* = 0.87.

**Fig. 1:**
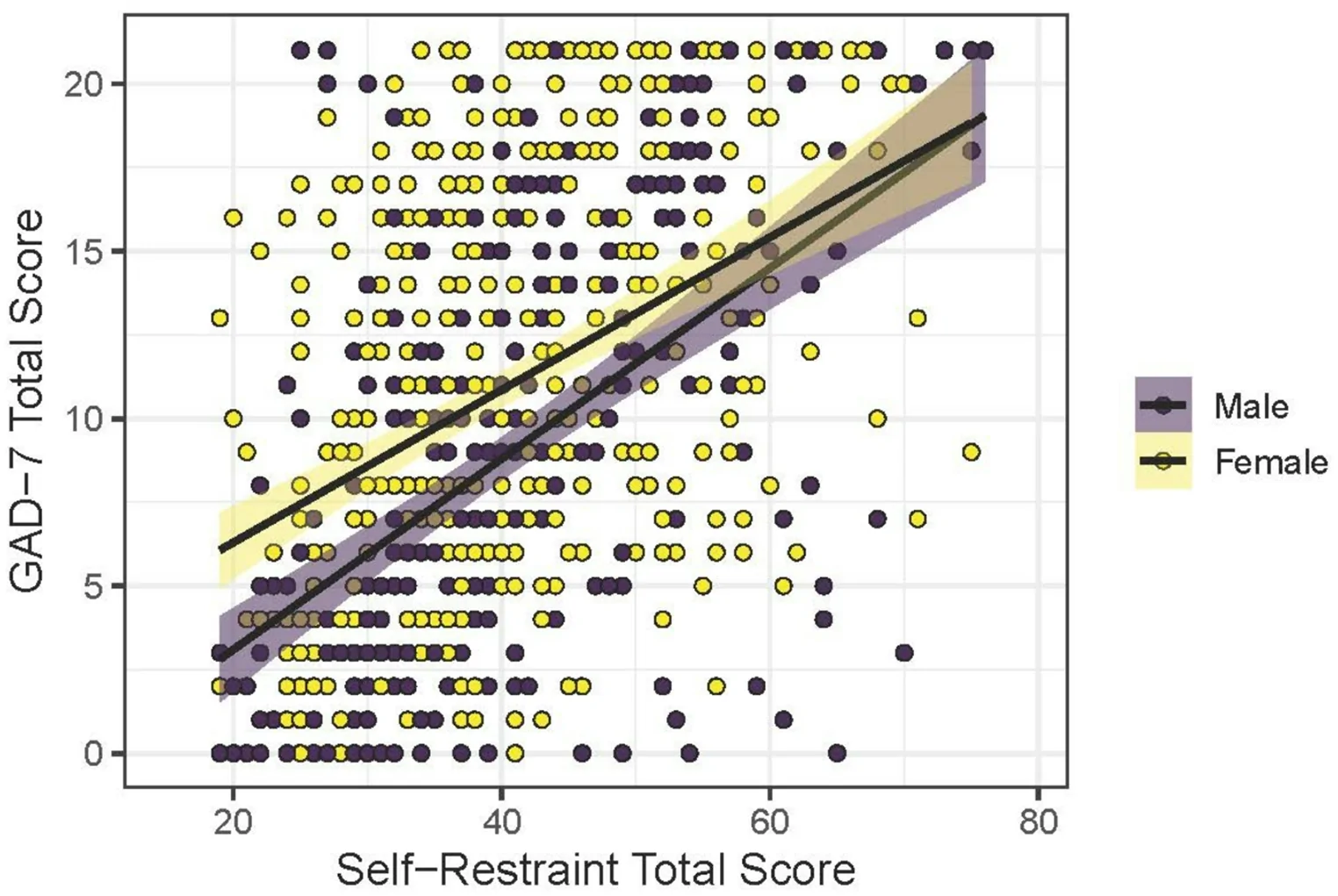
Association between inhibitory control challenges (BDEFS self-restraint) and anxiety symptomatology (GAD-7) by sex assigned at birth

Aim 3 sought to describe the relationship between inhibitory control challenges and anxiety symptoms. A hierarchical linear regression examining the relationship between Self-Restraint raw scores and GAD-7 total scores, with age entered in the first step as a covariate, was significant. It was found that inhibitory control challenges, as measured by the Self-Restraint raw score entered in step two, accounted for significant (20.4%) variance in generalized anxiety symptoms, even after accounting for age, Δ*F*(1,729) = 188.51, *p* <.001 (see Table 6). The effect size of the predictive model, measured by Cohen’s *f*^2^, was *f*^2^ = 0.27, indicating a medium effect. Sex assigned at birth was then added into the model in the third step and was shown to be a significant predictor of the GAD-7 total score, Δ*F*(1,728) = 23.41, *p* <.001, indicating that participants assigned female at birth endorsed more anxiety symptoms. The interaction effect between sex and Self-Restraint, entered in the fourth step, was not significant, Δ*F*(1,727) = 2.90, *p* =.089, suggesting that sex assigned at birth is not a significant moderator of the relationship between inhibitory control challenges and anxiety symptoms (see Fig. 1).

**Table 6:** Hierarchical regression model of GAD-7 total score with sex assigned at birth as potential moderator

Step 1: Age	*R*^2^ Δ = 0.009	*F* Δ(1, 730) = 6.963**
Step 2: Self-Restraint Raw Score	*R*^*2*^ Δ = 0.204	*F* Δ(1, 729) = 188.514***
Step 3: Sex Assigned at Birth (0 = Male; 1 = Female)	*R*^*2*^ Δ = 0.025	*F* Δ(1, 728) = 23.412***
Step 4: Sex * Self-Restraint Interaction	*R*^*2*^ Δ = 0.003	*F* Δ(1, 727) = 2.903
	*β* ^*1*^	*t*
Age	-0.68	-2.108*
Self-Restraint Raw Score	0.515	10.766***
Sex Assigned at Birth	0.348	2.982**
Sex * Self-Restraint Interaction	-0.207	-1.704

*p <.05. **p <.01. ***p <.001; 1: ***β*** values (and corresponding t-score) taken from final model, inclusive of all variables

Abbreviations: GAD-7 = Generalized Anxiety Disorder 7-item scale

**Fig. 2:**
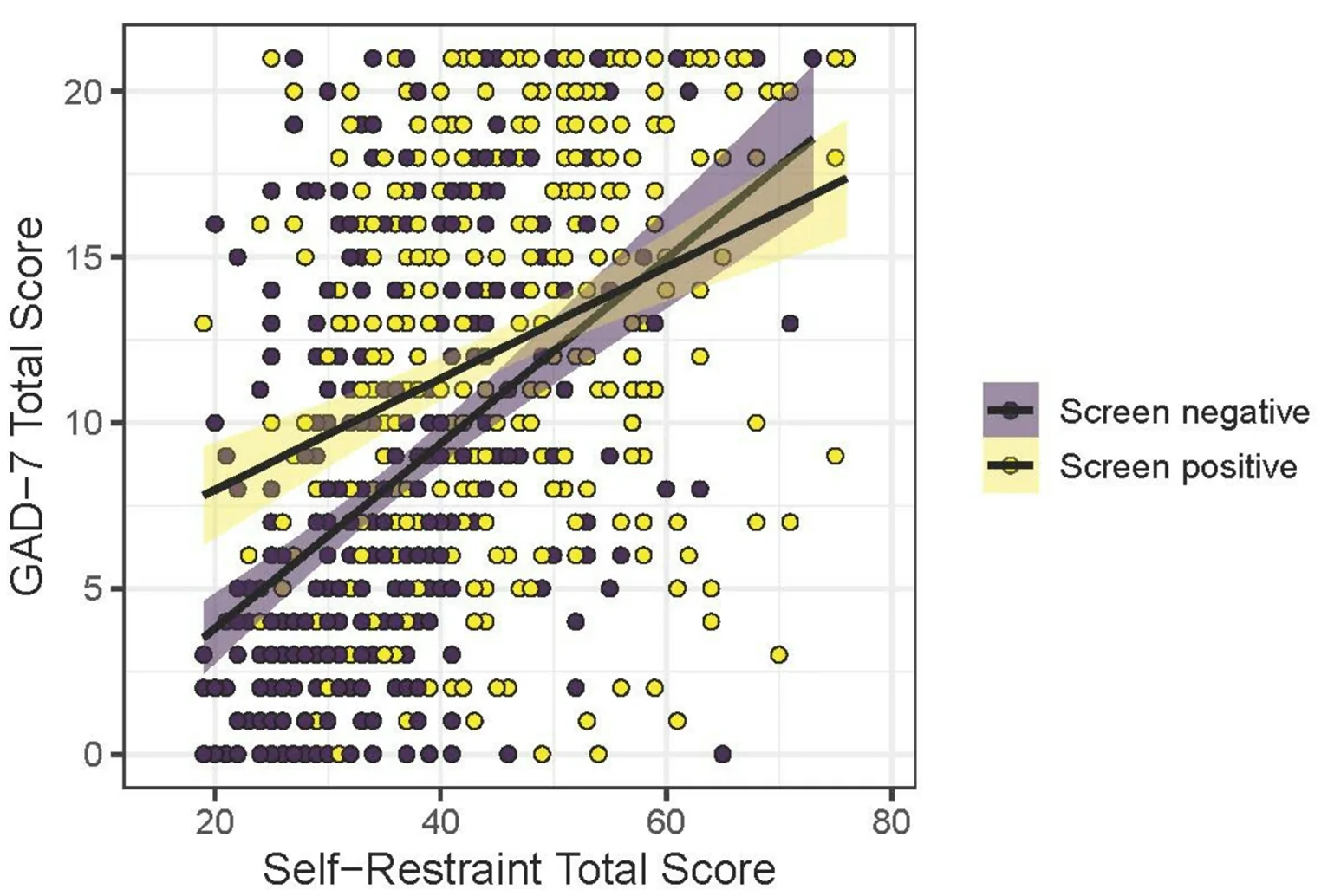
Association between inhibitory control challenges (BDEFS self-restraint) and anxiety symptomatology (GAD-7) by ADHD screening status

In the next model, we evaluated the moderating effect of ADHD screening status on the relationship between inhibitory control challenges and anxiety. Because sex assigned at birth was shown to be associated with anxiety symptomatology but did not moderate the relationship between inhibitory control challenges and anxiety symptomatology, it was included in this model as a covariate. For this model, the hierarchical regression included age and sex in the first step as covariates, the Self-Restraint raw scores in the second step, ADHD status in the third step, and the interaction between ADHD status and Self-Restraint in the last step (see Table 7). This model was significant, *F*(5,721) = 51.38, *p* <.001, and the effect size was *f*^*2*^ *=* 0.35, indicating a large effect. Each step added significant unique variance to the model, with the covariates contributing 3%, Self-Restraint contributing 20.7%, (Δ*F*(1,723) = 196.25, *p* <.001), ADHD screening status contributing 1.8% (Δ*F*(1,722) = 17.60, *p* <.001) and the interaction term contributing 0.7% (Δ*F*(1,721) = 7.24, *p* =.007). To help interpret the significant interaction effect, Pearson correlations were run separately for those who screened positive versus negative on the ADHD screener. An examination of the correlation coefficients revealed that the relationship between inhibitory control challenges and anxiety symptomatology was stronger in participants who screened negative for ADHD, *r*(375) = 0.45, *p* <.001 compared to those who screened positive, *r*(348) = 0.32, *p* <.001 (see Fig. 2). Fisher’s r-to-z transformation showed that the magnitude of the two correlations was significantly different, *z* = 2.05, *p =*.04.

**Table 7:** Hierarchical regression model of GAD-7 total score with suspected ADHD status as potential moderator

Step 1: Age & Sex Assigned at Birth	*R*^2^ Δ = 0.030	*F* Δ(2, 724) = 11.225***
Step 2: Self-Restraint Raw Score	*R*^*2*^ Δ = 0.207	*F* Δ(1, 723) = 196.249***
Step 3: ADHD Screener (0 = Negative; 1 = Positive)	*R*^*2*^ Δ = 0.018	*F* Δ(1, 722) = 17.603***
Step 4: ADHD * Self-Restraint Interaction	*R*^*2*^ Δ = 0.007	*F* Δ(1, 721) = 7.244**
	*β* ^*1*^	*t*
Age	-0.069	-2.14*
Sex Assigned at Birth	0.139	4.328***
Self-Restraint Raw Score	0.503	9.566***
Suspected ADHD Status	0.475	3.748***
ADHD * Self-Restraint Interaction	-0.392	-2.691**

*p <.05. **p <.01. ***p <.001; 1: ***β*** values (and corresponding t-score) taken from final model, inclusive of all variables

Abbreviations: GAD-7 = Generalized Anxiety Disorder 7-item scale

## Discussion

The present investigation sought to (1) characterize inhibitory control challenges in autistic adults; (2) explore the impact of sex assigned at birth and ADHD screening status on inhibition; (3) investigate the relationship between inhibitory control and anxiety; and (4) explore sex assigned at birth and ADHD screening status as moderators of the relationship between inhibition and anxiety. The results augment the limited existing knowledge base by showing statistically significant effects that have not been previously reported. Findings indicate that autistic adults experience significantly more challenges in inhibitory control than adults in the general population, and that individual differences in inhibitory control difficulties are related to anxiety symptomatology. The current study has several strengths, including a large sample size, inclusion of participants who have screened positive for ADHD on a validated screening tool, and the inclusion of a large group of autistic individuals assigned female at birth. Additionally, this study advances our understanding of executive function in autistic *adults*, who have been the focus of a limited number of research studies relative to autistic youth.

The results showed significantly increased self-reported challenges in inhibitory control in this sample of autistic adults relative to age- and assigned sex-based normative expectations. Existing literature has suggested that difficulties in inhibitory control likely persist into adulthood in autistic individuals (see Table 1), though many previous studies showed trends that did not reach statistical significance. Leveraging a large sample size, the current study was able to observe a large and statistically significant effect of self-reported inhibitory control challenges in autistic adults that has not been previously reported.

Participants in this study did not differ in raw inhibitory control scores on the basis of sex assigned at birth; however, a small but statistically significant difference in the sex- (and age-) adjusted standardized scores between individuals assigned male at birth and individuals assigned female at birth was observed, suggesting that autistic individuals assigned female may not benefit from the same sex-based inhibitory control advantage that is present in individuals assigned female at birth in the general population. This shows that, while there is no absolute difference in inhibitory control challenges between autistic individuals assigned male and autistic individuals assigned female, inhibitory control challenges may be more functionally impairing in autistic individuals assigned female, because they differ significantly from normative expectations for individuals assigned female from the general population. For autistic females, the sex-adjusted scores may reflect a more meaningful impact of inhibitory control challenges, because they may be expected (in their vocational activities or social relationships) to have relatively intact inhibitory control skills, as is the norm for their same-sex non-autistic peers. This is a noteworthy finding in the context of the current autism literature, which overwhelmingly samples autistic people assigned male at birth (D’Mello et al., 2022).

In concordance with existing research that shows significant inhibitory control challenges in individuals with ADHD (Chamorro et al., 2022; Willcutt et al., 2005), with and without co-occurring autism, the results of this investigation showed that autistic adults screening positive for ADHD endorsed significantly more inhibitory control challenges than autistic adults screening negative for ADHD. This finding, which suggests that ADHD is an important factor in the characterization of inhibitory control in autistic adults, highlights a need for more research on the role of co-occurring conditions, which are frequently excluded from (or not accounted for in) autism research, particularly during adulthood (Yerys, 2020; Yerys et al., 2022; Yerys et al., 2025). The subject of autism-ADHD co-occurrence has been the focus of recent research (e.g., Hours et al., 2022), which has emphasized the need for further exploration of symptom overlap and neurobiological etiologies of symptoms such as inhibitory control challenges. The current study’s finding that ADHD interacts with the endorsement of inhibitory control challenges in autistic adults demonstrates a need for research that explores the origins and manifestations of inhibitory control challenges in adults with co-occurring autism and ADHD. As highlighted by Hours and colleagues (2022), the overlapping yet distinct profiles of executive dysfunction in autism and ADHD complicate our understanding of shared symptoms. Incorporating these distinctions into future research may refine our understanding of inhibitory control challenges in autistic adults, particularly those with co-occurring ADHD symptoms, and improve intervention strategies tailored to this population.

This study also aimed to describe the relationship between inhibitory control challenges and anxiety symptoms through autistic adults’ self-report and to explore the potential moderating role of sex assigned at birth and ADHD screening status on this relationship. The results showed that inhibitory control challenges are significantly associated with anxiety symptoms after accounting for age and sex, such that greater endorsement of inhibitory control challenges was associated with greater anxiety symptoms. While previous research has suggested that executive function challenges broadly are associated with challenges in mental health (Wallace et al., 2016; Zimmerman et al., 2017) and quality of life (Dijkhuis et al., 2017) among autistic adults, there is limited research specifically investigating the relationship between inhibitory control challenges and anxiety. Only one previous study was identified that examined this relationship, and the results of that study showed a nonsignificant relationship between inhibitory control performance and anxiety symptoms (Zimmerman et al., 2017). The current study differs from this previous research in its large sample size and use of self-report measures, which may be more sensitive to everyday challenges in executive function. These differences may account for the differing findings in the two studies, and highlight the need for future research that examines both questionnaire and performance-based measures of inhibitory control.

ADHD screening status was found to be a significant moderator of the relationship between inhibitory control and anxiety, such that the relationship was strongest in participants who screened negative on the ADHD symptom inventory. The moderating role of ADHD symptomatology has not yet been explored in prior investigations of this relationship, so this finding presents an opportunity for further investigation. One possible explanation for this finding is that, among autistic adults who screen positive for ADHD, the extent of self-reported inhibitory control challenges may be more closely related to their ADHD symptoms (i.e., consistent with early theories of ADHD that highlight a primary deficit in inhibition; Barkley, 1997), and therefore related less to anxiety. Individuals who have a high number of inhibitory control challenges not in the presence of other ADHD symptoms, however, may find that their inhibitory control challenges are more related to aspects of thoughts and behavior that influence mental health, such as inhibiting negative thoughts that lead to anxiety symptoms. This explanation is speculative, but future research may benefit from examining this possibility.

Among the limitations of this study are the generalizability of findings to the broader autistic population. This research included participants who identified primarily as white (82.92%) and non-Hispanic (89.75%), limiting conclusions that can be made about the nature of inhibitory control and anxiety symptoms in the broader autistic population. Another limitation to consider is that this sample is not inclusive of individuals with co-occurring intellectual disability, which impacts approximately one third of autistic individuals (Maenner et al., 2023). This further limits the generalizability of findings to all autistic adults.

Additionally, the current study used all self-report measures. While the inclusion of self-reported challenges can give unique insight into day-to-day challenges in executive function and mental health domains, future research in this area should explore whether these results persist when using performance-based executive function tasks in conjunction with self-report and/or clinician ratings of anxiety. Another limitation of this research is the cross-sectional study design. This design prevents discussion of directionality or causality in the relationship between inhibitory control and anxiety. Future research should aim to investigate how changes to inhibitory control over time are related to mental health.

In addition, this study employed a screener of ADHD symptoms and did not consider community-based ADHD diagnoses. This choice was made in order to capture ADHD symptoms in autistic adults who may have had limited access to a formal community diagnosis (Eberhard et al., 2022; Magnin & Maurs, 2017; Martin et al., 2024). Given that the results suggest that co-occurring ADHD may play a role in the occurrence of inhibitory control difficulties and their relationship with psychopathology in autistic adults, future studies should explore the moderating role of ADHD as assessed via a formal diagnostic evaluation. This study was also not able to confirm autism spectrum diagnostic status, which may be considered a limitation of the study design. However, as mentioned previously, many SPARK participants are recruited from expert autism clinical sites, increasing the likelihood that participants have a professionally assigned autism diagnosis. Additionally, previous research on the SPARK database confirmed, with information from participants’ electronic medical records, that 98.8% of a sample of SPARK participants indeed had a documented autism spectrum diagnosis (Fombonne et al., 2022). Finally, this study did not control for other potentially co-occurring psychiatric diagnoses which could account for differences in inhibitory control or anxiety symptoms or explain their relationship.

Acknowledging these limitations, the results of this investigation may have implications for the assessment and treatment of mental health conditions in autistic adults. The identification of individual risk factors for increased anxiety, such as inhibitory control difficulties, is important for evaluating factors that may relate to heterogeneity in outcomes among autistic adults as well as the possible moderating effect of poor inhibitory control on treatment responsiveness. For example, traditional cognitive behavioral therapy (CBT) for anxiety symptomatology asks individuals to observe and interrupt their automatic negative thoughts; recognizing that autistic adults with inhibitory control challenges may struggle to halt their automatic thoughts, it may be helpful for clinicians to consider whether additional supports are needed when conducting CBT with autistic adults with inhibitory control challenges. In pursuit of informing clinical care, future research should aim not only to replicate the findings from the current study but also evaluate if inhibitory control challenges moderate treatment effectiveness. Understanding factors that may underlie differences in mental health may help identify individuals more at risk for suboptimal outcomes as well as alternative treatment targets for future intervention research.

## Footnotes

1 We use identity-first language (e.g., “autistic individuals” or “autistic adults”) throughout this research project as this term is preferred by autistic self-advocates (Botha et al., 2020, 2023; Kenny et al., 2016; Vivanti, 2020).
